# A cell-penetrating PHLPP peptide improves cardiac arrest survival in murine and swine models

**DOI:** 10.1172/JCI164283

**Published:** 2023-05-01

**Authors:** Jing Li, Xiangdong Zhu, Matt T. Oberdier, Chunpei Lee, Shaoxia Lin, Sarah J. Fink, Cody N. Justice, Kevin Qin, Andrew W. Begeman, Frederick C. Damen, Hajwa Kim, Jiwang Chen, Kejia Cai, Henry R. Halperin, Terry L. Vanden Hoek

**Affiliations:** 1 Center for Advanced Resuscitation Medicine, Department of Emergency Medicine, Center for Cardiovascular Research, University of Illinois at Chicago, Chicago, Illinois, USA.; 2 Department of Medicine, Johns Hopkins University, Baltimore, Maryland, USA.; 3 Department of Radiology, College of Medicine,; 4 Center for Clinical and Translational Science,; 5 Cardiovascular Research Center, and; 6 Department of Medicine, Division of Pulmonary, Critical Care, Sleep and Allergy, University of Illinois at Chicago, Chicago, Illinois, USA.; 7 Departments of Radiology and Biomedical Engineering, Johns Hopkins University, Baltimore, Maryland, USA.

**Keywords:** Therapeutics, Cardiovascular disease, Drug therapy, Peptides

## Abstract

Out-of-hospital cardiac arrest is a leading cause of death in the US, with a mortality rate over 90%. Preclinical studies demonstrate that cooling during cardiopulmonary resuscitation (CPR) is highly beneficial, but can be challenging to implement clinically. No medications exist for improving long-term cardiac arrest survival. We have developed a 20–amino acid peptide, TAT-PHLPP9c, that mimics cooling protection by enhancing AKT activation via PH domain leucine-rich repeat phosphatase 1 (PHLPP1) inhibition. Complementary studies were conducted in mouse and swine. C57BL/6 mice were randomized into blinded saline control and peptide-treatment groups. Following a 12-minute asystolic arrest, TAT-PHLPP9c was administered intravenously during CPR and significantly improved the return of spontaneous circulation, mean arterial blood pressure and cerebral blood flow, cardiac and neurological function, and survival (4 hour and 5 day). It inhibited PHLPP-NHERF1 binding, enhanced AKT but not PKC phosphorylation, decreased pyruvate dehydrogenase phosphorylation and sorbitol production, and increased ATP generation in heart and brain. TAT-PHLPP9c treatment also reduced plasma taurine and glutamate concentrations after resuscitation. The protective benefit of TAT-PHLPP9c was validated in a swine cardiac arrest model of ventricular fibrillation. In conclusion, TAT-PHLPP9c may improve neurologically intact cardiac arrest survival without the need for physical cooling.

## Introduction

Out-of-hospital cardiac arrest (CA) affects 350,000 people annually, with an overall survival rate of less than 10% (1). Cardiopulmonary resuscitation (CPR), defibrillation, and targeted temperature management (TTM) using active cooling are the few effective treatment strategies that improve survival. Despite the return of spontaneous circulation (ROSC), most patients die within hours due to cardiovascular collapse, impaired metabolic recovery of vital organs, brain injury, and systemic inflammation after CA (2). Two critical organs that determine the outcomes of post-CA patients are the heart and the brain.

When external cooling (32°C to 36°C) was applied to comatose but hemodynamically stable CA patients, neurologically intact survival improved by 20% (3, 4). Although TTM (active cooling initiated after ROSC to a target temperature of 32°C to 36°C) has shown benefit, it can be difficult to implement and the optimal target temperature and duration remain unknown. In preclinical studies, intra-CPR cooling to 30°C to 34°C (cooling initiated prior to ROSC during CPR) had more profound therapeutic potential than cooling after ROSC (5, 6). However, it is difficult to achieve clinically. An ideal alternative would be a medication that could be delivered rapidly to reproduce the protection without the need for physical cooling. The recently published TTM2 trial (Targeted Hypothermia Versus Targeted Normothermia after Out-of-Hospital Cardiac Arrest) included a large. diverse population of out-of-hospital CA patients across Australia, Europe, and the US and demonstrated that cooling at 33°C did not result in significant differences in 6-month survival as compared with 37°C, raising concerns regarding the benefits of TTM delivered after ROSC (7). Therefore, a therapeutic strategy that provides rapid access to tissues after CA during CPR to improve survival would represent a substantial advancement in resuscitation medicine.

We and others demonstrated that CPR cooling protection is mediated by AKT activation, a key cellular regulator for survival, metabolism, and inflammation (8, 9). In cardiomyocytes, we showed that ischemia/reperfusion (I/R) injury is markedly reduced by intraischemic cooling prior to reperfusion. This protection is blocked by the AKT inhibitor API-2 (10). Furthermore, intra-CPR cooling protection was lost in *Akt1^+/–^* mice (11, 12), highlighting a critical role for AKT. Additional work using a small molecule inhibitor of phosphatase and tensin homolog deleted on chromosome ten (PTEN) and a PTEN-inhibitory peptide induced marked activation of AKT in mouse after CA, with significantly decreased inflammation, increased metabolic recovery, and improved survival (11, 13). However, optimal protection by the small molecule inhibitor required pretreatment. We took a further step by developing biological AKT-related phosphatase inhibitors that are more rapid acting and potent. PH domain leucine-rich repeat phosphatase 1 (PHLPP1) is an abundant phosphatase that dephosphorylates AKT at the hydrophobic motif Ser473, one of the two major phosphorylation sites (Thr308 and Ser473) required for AKT activation (14, 15). Cardiomyocytes with *Phlpp1* knockdown or deficiency demonstrate heart protection against doxorubicin and H_2_O_2_-induced injury (15). *Phlpp1*-knockout mice demonstrated increased AKT activation and brain protection against I/R injury after middle cerebral artery occlusion (16). Therefore, we have developed a 20–amino acid cell-permeable peptide, TAT-PHLPP9c, that enhances AKT activity via PHLPP1 inhibition. This peptide contains 9 C-terminal residues of PHLPP1 and 11 amino acids of the cell-membrane transduction domain of the TAT protein. It selectively inhibits the ability of PHLPP1 to dephosphorylate AKT. We hypothesized that TAT-PHLPP9c improves neurologically intact survival after CA when administered intravenously during CPR through enhancing glucose oxidation after resuscitation.

## Results

### TAT-PHLPP9c peptide activity and its mechanism of action.

**Figure 1:**
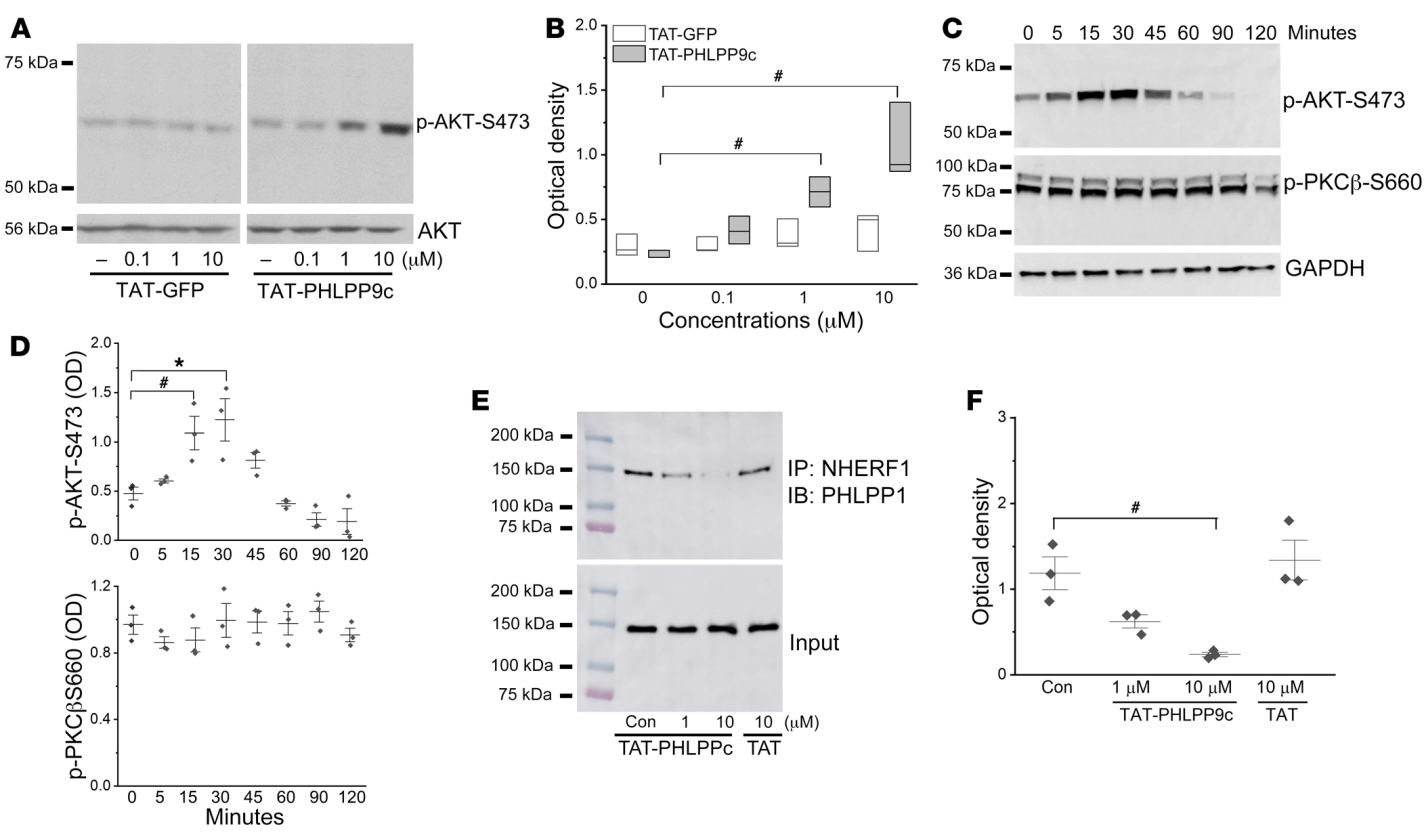
TAT-PHLPP9c activity and the mechanism of action. (**A**) TAT-PHLPP9c induced AKT phosphorylation at Ser473 in a dose-dependent manner (0.1, 1, 10 μM) in mouse ventricular cardiomyocytes. Total AKT was used as a loading control. (**B**) Densitometric analysis of AKT phosphorylation. One-way ANOVA with Dunnett’s test was used. ^#^*P* < 0.05 between TAT-GFP control and TAT-PHLPP9c (at 1 μM and 10 μM). Data are represented as mean ± SD for 3 experiments. (**C**) TAT-PHLPP9c (10 μM) increased AKT phosphorylation in a time-dependent manner with a peak at 30 minutes in cardiomyocytes. It had no effect on PKCβ2-S660 phosphorylation. GAPDH was used as a loading control. (**D**) Densitometric analysis of AKT and PKCβ2-S660 phosphorylation in response to TAT-PHLPP9c treatment. One-way ANOVA with Dunnett’s test was used. ^#^*P* < 0.05 for time 0 to 15 minutes with TAT-PHLPP9c (10 μM); *P < 0.01 for time 0 to 30 minutes with TAT-PHLPP9c. Data are represented as mean ± SD for 3 experiments. (**E**) TAT-PHLPP9c blocked endogenous PHLPP1 binding to its membrane adaptor NHERF1. Mouse brain lysates (500 μg) were incubated with TAT-PHLPP9c (1 and 10 μM) or 10 μM of TAT for 30 minutes and were precipitated with NHERF1 antibody; immunoblots were analyzed using antibody against PHLPP1. Equal input into the immunoprecipitation reaction was verified by PHLPP1 antibody. TAT-PHLPP9c at 1 to 10 μM, but not TAT vehicle, inhibited endogenous PHLPP1 binding to NHERF1. (**F**) Densitometric analysis of immunoprecipitation assay of PHLPP1 and NHERF1 interaction. Paired, 2-tailed *t* test was used. ^#^*P* < 0.05 between control and 10 μM of TAT-PHLPP9c; *P* = 0.51 between control and 10 μM of TAT. Data are represented as mean ± SD for 3 experiments.

We first determined whether TAT-PHLPP9c enhanced AKT activation in primary mouse cardiomyocytes. Cardiomyocytes were treated with various concentrations of TAT-PHLPP9c (0.1, 1, and 10 μM) or equal concentrations of TAT vehicle control for 15 minutes and AKT phosphorylation at Ser473 was measured. Compared with the TAT control, TAT-PHLPP9c increased AKT-Ser473 phosphorylation (at 1 and 10 μM, *P* < 0.05) in a dose-dependent manner (Figure 1, A and B). TAT-PHLPP9c (10 μM) induced time-dependent AKT phosphorylation. It significantly increased AKT-Ser473 phosphorylation at 15 minutes after treatment and peaked at 30 minutes in cardiomyocytes. It had no effect on PKC phosphorylation measured by PKCβ2-S660, a phosphorylation site regulated by PHLPP 1 and shared by PKCα, -β1, -β2, -δ, -ε, -η, and -θ isoforms (Figure 1, C and D). TAT-PHLPP9c at 1 to 10 μM, but not TAT vehicle, inhibited endogenous PHLPP1 binding to its membrane adaptor Na^+^/H^+^ exchanger regulatory factor 1 (NHERF1), as revealed by immunoprecipitation assay (Figure 1, E and F).

### Kinetics of TAT-protein delivery to heart and brain.

**Figure 2:**
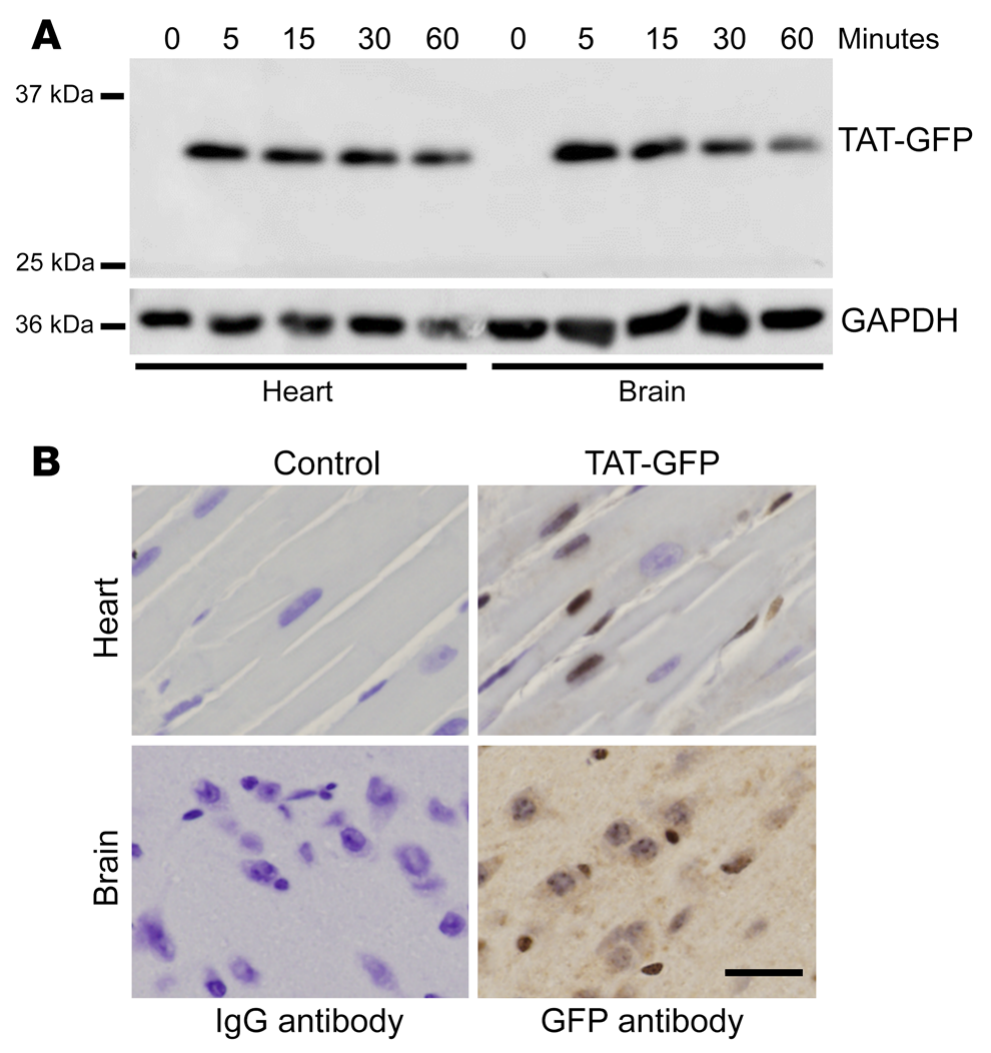
TAT fusion protein expression in mouse tissues. (**A**) TAT-GFP (7.5 mg/kg) was administered via intravenous injection. Heart and brain tissues were collected at various time points after injection (5, 15, 30, and 60 minutes) and measured for GFP expression by Western blot. TAT-GFP expression was detected in both heart and brain as quickly as 5 minutes after injection and gradually decreased over time. GAPDH was used as a loading control. (**B**) Immunohistochemistry analysis of TAT-GFP 60 minutes after intraperitoneal injection. Mouse heart and brain were stained with isotype control or anti-GFP antibody (brown) and counterstained with hematoxylin. Scale bar: 50 μM. Data represent *n* = 3 mice.

We next examined the kinetics of TAT-peptide delivery in brain and heart tissues of naive mice. GFP-conjugated TAT protein (7.5 mg/kg) was administered intravenously. Heart and brain tissue were harvested after 0, 5, 10, 30, and 60 minutes of injection and measured for GFP expression. As depicted in Figure 2A, TAT-GFP was detected in both heart and brain within 5 minutes of administration. It gradually decreased over time and was detectable up to 60 minutes after injection. We also observed that TAT-GFP could be detected in both the cytosol and nucleus of heart and brain tissues 60 minutes after injection, as illustrated by immunohistochemistry staining (brown staining), compared with control IgG (Figure 2B). These results demonstrated a rapid delivery of TAT protein to vital organs after administration that lasted at least 60 minutes.

### Efficacy of TAT-PHLPP9c on improving 4-hour mouse survival after CA.

**Figure 3:**
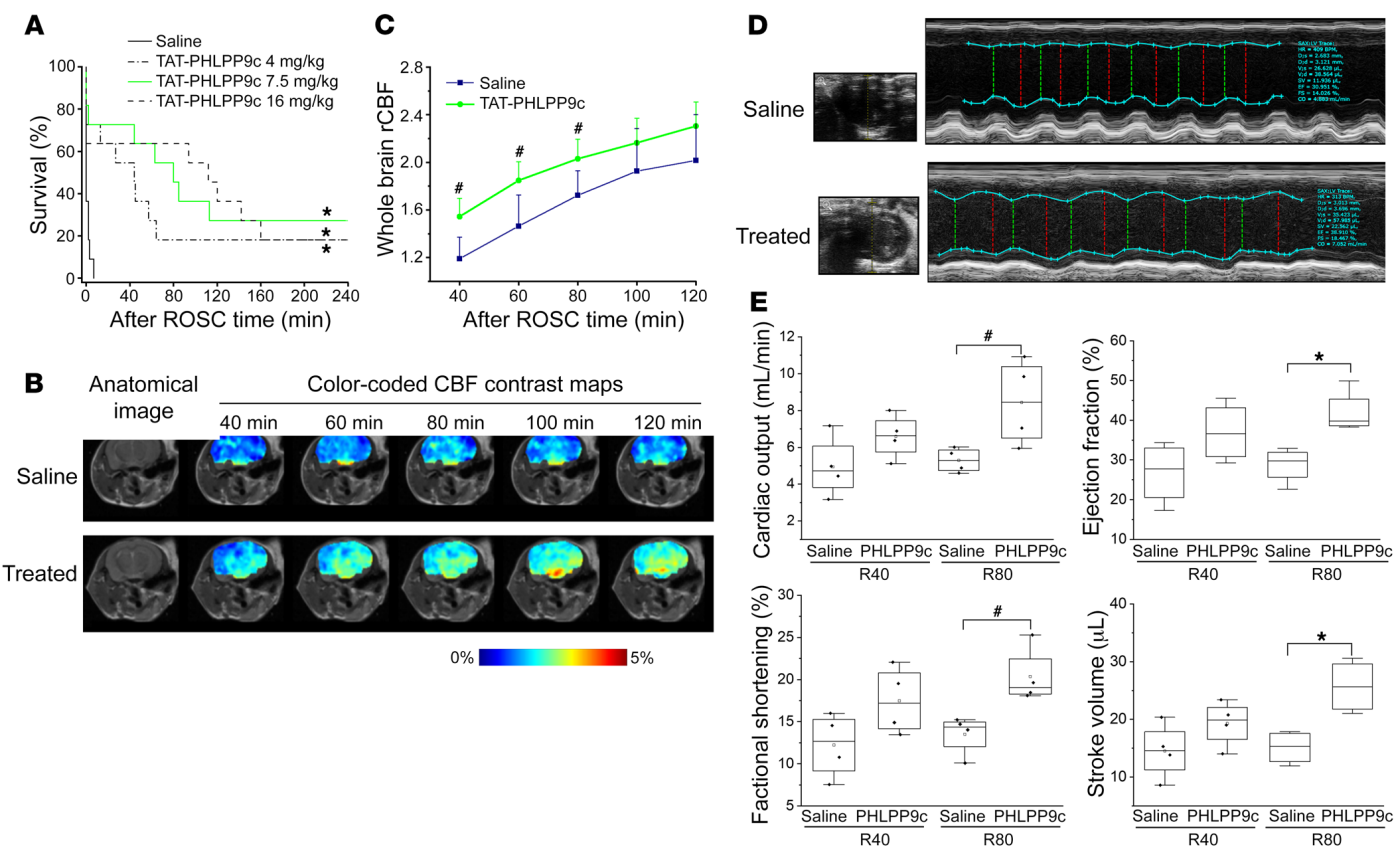
TAT-PHLPP9c improved 4-hour survival, brain perfusion, and cardiac function when administered during CPR after mouse CA. (**A**) Kaplan-Meier 4-hour survival plot demonstrated that, compared with saline, TAT-PHLPP9c administered at various doses (4. 7.5 and 16 mg/kg) improved 4-hour survival following 12 minutes of arrest, with 7.5 mg/kg being a lower dose with highest benefit. No significant difference between the doses of 7.5 and 16 mg/kg. *n* = 11 in each group. A log-rank (Mantel-Cox) test using Kaplan-Meier curves was performed. *P < 0.01 between saline and TAT-PHLPP9c. (**B**) Longitudinal MRI revealed that TAT-PHLPP9c treatment significantly increased CBF following 8 minutes of arrest. Anatomical T_2_-weighted MRI images of the brain and the color-coded CBF contrast maps at varied time points after ROSC (40, 60, 80, 100, and 120 minutes) overlaid onto the anatomical T_2_-weighted images from a representative control mouse (top) and a treated mouse (bottom). (**C**) Statistical analysis of rCBF MRI contrast over time from saline (black) and TAT-PHLPP9c (green). Paired, 2-tailed *t* test was used. ^#^*P* < 0.05. Data are represented as mean ± SD for 5 mice. (**D**) Representative echocardiography SAX images revealed that TAT-PHLPP9c improved heart ventricular contraction after ROSC. SAX images of cross-sectional view of left and right ventricles captured at R80 after ROSC from a representative control mouse (top) and a treated mouse (bottom). (**E**) Compared with saline, TAT-PHLPP9c improved cardiac function, including CO, EF, FS, and SV. The improvement was significant at R80. Paired, 2-tailed *t* test was used. ^#^*P* < 0.05; *P < 0.01. Data are represented as mean ± SD for 4 mice.

The efficacy of TAT-PHLPP9c in a mouse CA model was tested with a bolus of TAT-PHLPP9c (7.5 mg/kg) given intravenously during CPR. ROSC rate and time, mean arterial blood pressure (MAP), and 4-hour survival were assessed. Baseline (BL) characteristics, including weight, heart rate, and MAP, were indistinguishable between the control and treatment groups. Resuscitation parameters were affected by TAT-PHLPP9c treatment. As depicted in Figure 3A, in the control group, 4 out of 11 mice achieved ROSC (36%), but they died quickly, within 10 minutes (R10) after ROSC. In contrast, 9 out of 11 mice achieved ROSC (82%) in the treatment group (*P* < 0.05). Among them, most lived longer than those in the control group and 3 mice lived to 4 hours (*P* < 0.01). These results demonstrated that TAT-PHLPP9c improved not only ROSC rates, but also increased 4-hour survival among ROSC mice to 30% after a prolonged CA. TAT-PHLPP9c demonstrated a statistically insignificant reduction in ROSC time (180.8 ± 48.3 seconds in control versus 159.3 ± 23.0 in treatment; *P* = 0.299). MAP could not be compared, as none of the mice in the saline group lived beyond 10 minutes after ROSC. We next examined the dose-response relationship. Two other doses of the peptide (4 and 16 mg/kg) were tested (Figure 3A). TAT-PHLPP9c at 4 mg/kg improved survival over that of controls (*P* < 0.01), but was less effective than 7.5 mg/kg. The protective effect was equivalent in doses of 16 mg/kg and 7.5 mg/kg. Therefore, the dose of 7.5 mg/kg presented the most efficacious effects, and it was determined to be the efficacy dose for the remaining studies. In addition, saline, instead of TAT-PHLPP3a (lacking binding capability to its membrane adaptor), was used as control for this study, as prior work showed that a peptide with a structure and design similar to that of TAT-PHLPP3a showed no significant differences following saline and control peptide administration on AKT phosphorylation, heart function, and survival in cardiomyocytes, isolated rat hearts, and a whole mouse CA model, respectively (13).

### TAT-PHLPP9c improved cerebral blood flow and cardiac function.

Brain perfusion recovery was evaluated by brain MRI measuring relative cerebral blood flow (rCBF) at various time points from 40 to 120 minutes following ROSC. We used a less severe mouse CA model (8 minutes of arrest instead of 12 minutes of arrest in other studies) to ensure rCBF post-ROSC images could be collected in both control and peptide-treated mice. Figure 3B illustrates representative longitudinal MRI images captured in saline- and peptide-treated mice. Color-coded brain rCBF maps revealed progressively increased signals in both control and treated mice, demonstrating continuous recovery of CBF after ROSC in both groups. Treated mice showed consistently higher rCBF signals than control mice at any given time point, demonstrating the benefit of the TAT-PHLPP9c peptide for improving whole-brain CBF. Figure 3C shows the longitudinal recovery curves of rCBF in both groups over time. Compared with what occurred in saline controls, the peptide increased CBF at all time points, with statistically significant differences observed at R40, R60, and R80 after ROSC (*P* < 0.05).

Cardiac function was measured by echocardiogram to assess systolic and diastolic function at R40 and R80 after ROSC. Figure 3D illustrates representative echocardiography parasternal short-axis (SAX) M-mode images of cross-sectional views of right and left ventricles captured in saline- and peptide-treated mice. As depicted in Figure 3E, TAT-PHLPP9c increased systolic function, including cardiac output (CO), ejection fraction (EF), fractional shortening (FS), and stroke volume (SV), although these changes were not statistically significant at R40 (Supplemental Table 1; supplemental material available online with this article; https://doi.org/10.1172/JCI164283DS1), possibly due to a small sample size. The improvement caused by TAT-PHLPP9c was significant at R80 after ROSC for CO (5.30 ± 0.66 mL/min in saline versus 8.44 ± 2.33 mL/min in TAT-PHLLP9c, *P* < 0.05), EF (28.79% ± 4.4% in saline versus 41.95% ± 5.42% in TAT-PHLLP9c, *P* < 0.01), FS (13.51% ± 2.33% in saline versus 20.37% ± 3.34% in TAT-PHLLP9c, *P* < 0.05), and SV (15.11 ± 2.91 mL in saline versus 25.71 ± 4.63 in TAT-PHLLP9c, *P* < 0.01). The diastolic function parameters, such as the E′/E ratio, E′/A′ ratio, isovolumic relaxation time (IVRT), and isovolumic contraction time (IVCT), showed no significant differences between saline and TAT-PHLPP9c.

### TAT-PHLPP9c increased AKT activation in both heart and brain.

**Figure 4:**
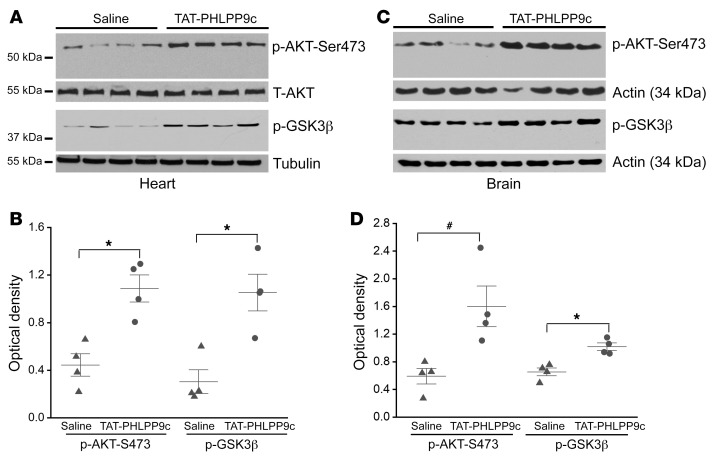
TAT-PHLPP9c increased phosphorylation of AKT and GSK3β analyzed by Western blot at R15 after ROSC in heart and brain. (**A**) p-AKT-Ser473 and p-GSK3β-Ser9 at R15 in heart. Total AKT and α-tubulin were used as loading controls for heart p-AKT-Ser473 and p-GSK3β-Ser9, respectively. (**B**) Densitometric analysis of Western blot of p-AKT-Ser473 and p-GSK3β-Ser9 in heart. Paired, 2-tailed *t* test was used. *P < 0.01 between saline control and TAT-PHLPP9c. Data are represented as mean ± SD for 4 mice. (**C**) p-AKT-Ser473 and p-GSK3β-Ser9 at R15 in brain. β-Actin was used as a loading control. (**D**) Densitometric analysis of p-AKT-Ser473 and p-GSK3β-Ser9 in brain. Paired, 2-tailed *t* test was used. ^#^*P* < 0.05 between saline and TAT-PHLPP9c; *P < 0.01 between saline and TAT-PHLPP9c. Data are represented as mean ± SD for 4 mice.

To confirm whether TAT-PHLPP9c increased AKT activation, heart and brain tissues were collected at R15 and measured for the phosphorylation of AKT and its downstream target, GSK3β. Compared with what occurred with saline control, the phosphorylation of AKT-Ser473 and GSK3β-Ser9 was significantly increased with the treatment of TAT-PHLPP9c in both heart (p-AKT-Ser473, *P* < 0.01; p-GSK3β-Ser9, *P* < 0.01) (Figure 4, A and B) and brain (p-AKT-Ser473, *P* < 0.05; p-GSK3β-Ser9, *P* < 0.01) (Figure 4, C and D).

### TAT-PHLPP9c improved glucose utilization.

**Figure 5:**
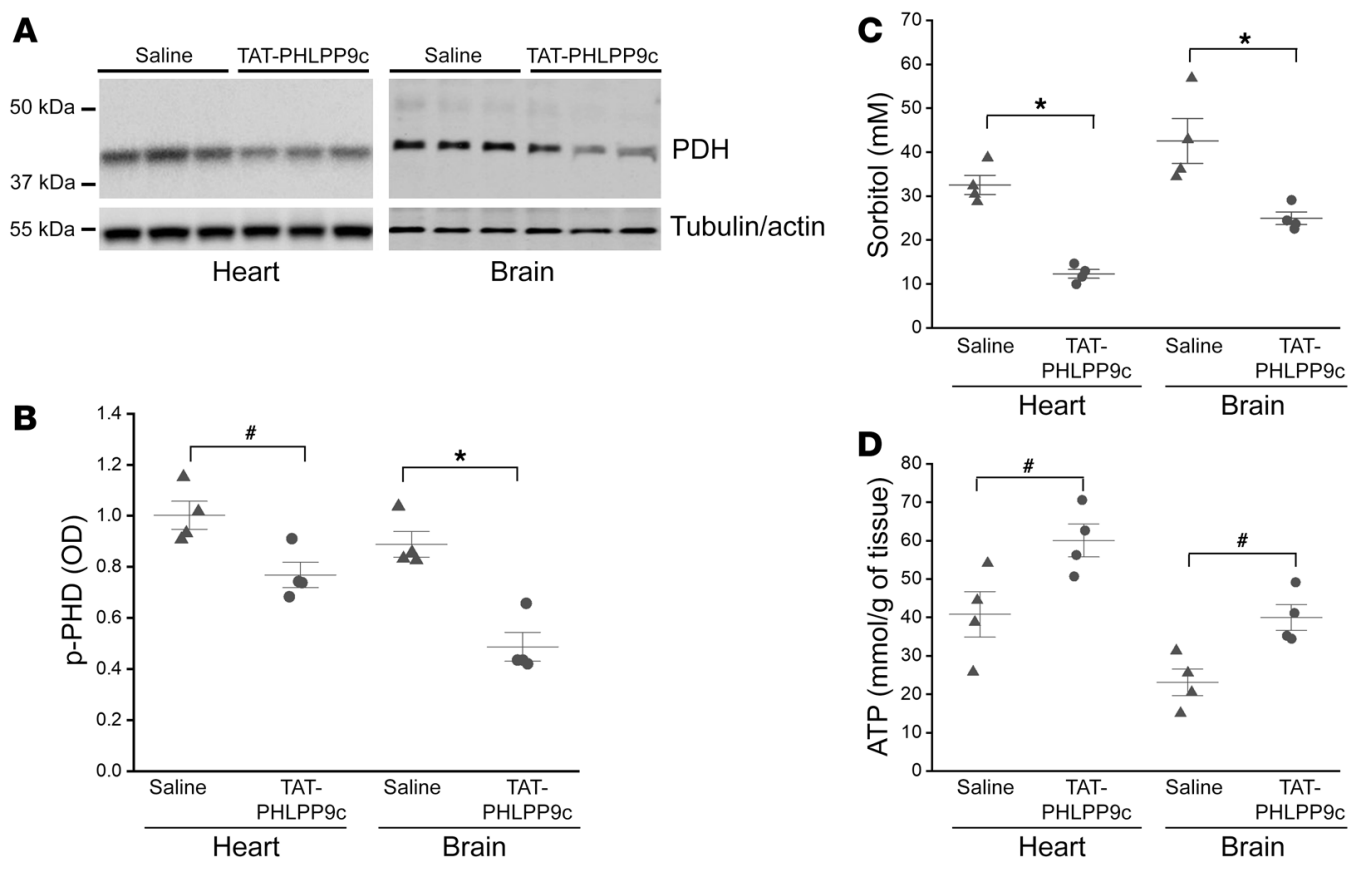
TAT-PHLPP9c decreased PDH phosphorylation, reduced sorbitol generation, and increased ATP content at R15 after ROSC in heart and brain. α-Tubulin and β-actin were used as loading controls for heart and brain, respectively. (**A**) Representative PDH phosphorylation at R15 in heart and brain. (**B**) Densitometric analysis of PDH phosphorylation in heart and brain. Paired, 2-tailed *t* test was used. ^#^*P* < 0.05 between saline control and TAT-PHLPP9c; *P < 0.01 between saline and TAT-PHLPP9c. Data are represented as mean ± SD for 4 mice. (**C**) Sorbitol contents at R15 in heart and brain. Paired, 2-tailed *t* test was used. *P < 0.01 between saline and TAT-PHLPP9c. Data are represented as mean ± SD for 4 mice. (**D**) ATP contents at R15 in heart and brain. Paired, 2-tailed *t* test was used. ^#^*P* < 0.05 between saline and TAT-PHLPP9c. Data are represented as mean ± SD for 4 mice.

Ischemic conditions, as seen in CA, trigger an immediate switch from aerobic metabolism to anaerobic metabolism. Ischemic tissues become critically dependent on glucose utilization for recovery during reperfusion. As a result of increased glycolysis, pyruvate is markedly increased. Pyruvate dehydrogenase (PDH) is a rate-limiting enzyme that shunts pyruvate to mitochondria for oxidation. Decreased PDH phosphorylation reflects increased PDH activity. Glucose can also be converted to sorbitol through the polyol pathway during ischemic injury, thereby increasing osmotic stress and tissue damage (13). Increased PDH activity increases shunting of pyruvate to the mitochondrion and reduces diversion of glucose to sorbitol generation, promoting glucose oxidation and ATP production. For this reason, we measured PDH phosphorylation at PDH E1-α subunit (p-Ser293), sorbitol, and ATP contents in heart and brain tissues collected at R15. The same heart and brain tissue samples used for the measurement of AKT and GSK3β phosphorylation were used. Compared with saline, TAT-PHLPP9c significantly decreased PDH-Ser293 phosphorylation in both heart and brain tissues (*P* < 0.01) (Figure 5, A and B). Sorbitol concentrations were also reduced by TAT-PHLPP9c, from 35.95 ± 8.49 μM to 11.35 ± 2.75 μM (*P* < 0.01) in heart and from 42.55 ± 10.19 μM to 32.27 ± 13.19 μM (*P* < 0.01) in brain (Figure 5C). Accordingly, ATP production was increased from 31.3 ± 2.34 μmol/g in control to 37.9 ± 2.6 μmol/g in heart (*P* < 0.05) and from 36.1 ± 3.66 μmol/g in control to 48.6 ± 5.6 μmol/g in brain (*P* < 0.05) (Figure 5D).

### TAT-PHLPP9c improved hemodynamic and neurologically intact 5-day survival.

**Figure 6:**
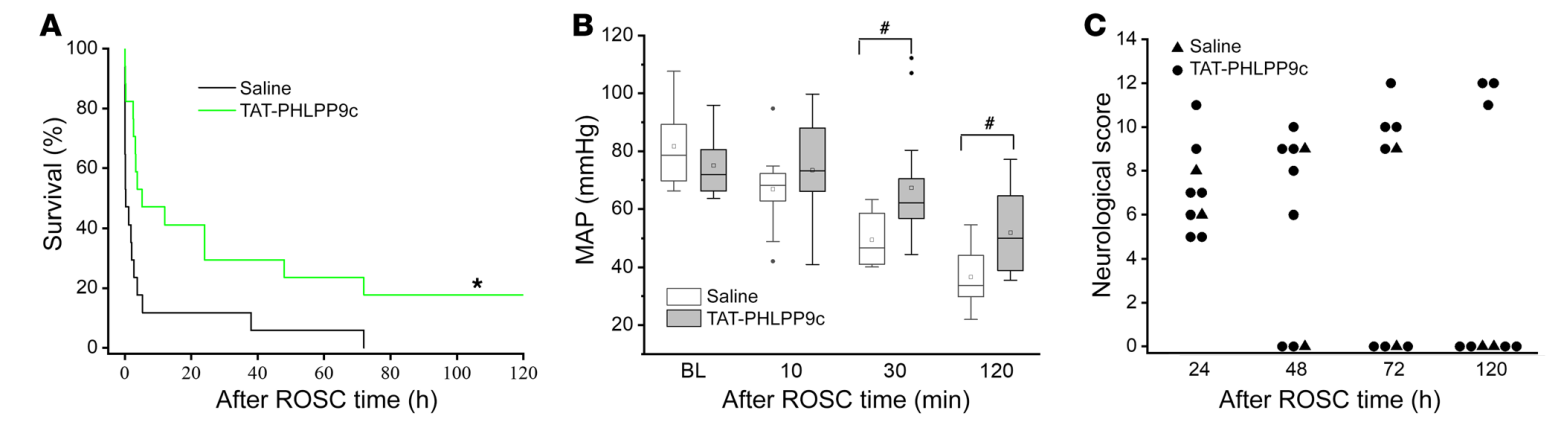
Effect of TAT-PHLPP9c on 5-day survival, MAP, and neurological function after CA in mouse. (**A**) Kaplan-Meier 5-day survival plot of saline control and TAT-PHLPP9c groups (*n* = 17 in each group). A log-rank (Mantel-Cox) test using Kaplan-Meier curves was performed. *P < 0.01 between saline and TAT-PHLPP9c. (**B**) Box plot of MAP in the surviving mice over time (BL, R10, R30, and R120). Compared with saline, TAT-PHLPP9c improved MAP at 30 minutes and 2 hours. Paired, 2-tailed *t* test was used. ^#^*P* < 0.05 between saline and TAT-PHLPP9c. Data are represented as mean ± SD. (**C**) Assessment of neurological function score (0 representing death of the animal and 12 reflecting a full neurological recovery) at 5 days after ROSC.

A randomized and blinded 5-day (120 hours) survival study was performed to further examine the effectiveness of TAT-PHLPP9c on long-term survival after CA and the recovery of neurological function. Two groups (saline control and TAT-PHLPP9c at 7.5 mg/kg) consisting of 17 mice in each group were included. As depicted in Figure 6A, survival was significantly improved with TAT-PHLPP9c treatment (*P* < 0.01). Two mice survived to 6 hours in the saline group compared with 7 in the treatment group. MAP was not noticeably different at BL, but was consistently higher in the treatment group than in the control group at R30 and R120 (*P* < 0.05) (Figure 6B). In the treatment group, neurological function continued to improve from 24 hours after ROSC (R24h) to R120h (Figure 6C). Only one mouse in the saline group survived to R72h with a fair neurological score in comparison with 4 mice in the peptide group, with 3 of them surviving to 5 days with normal or near-normal neurological scores (Figure 6C).

### Confirmation of efficacy of TAT-PHLPP9c in a swine ventricular fibrillation CA model.

**Figure 7:**
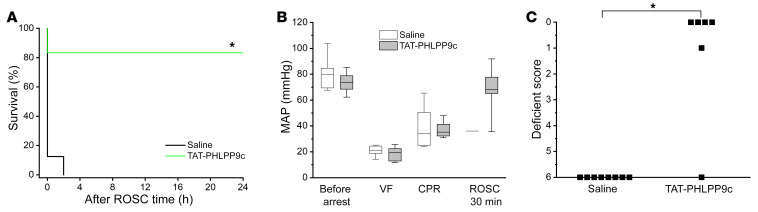
Effect of TAT-PHLPP9c on 24-hour survival, MAP, and neurological function after CA in swine. (**A**) Kaplan-Meier 24-hour survival plot of saline and TAT-PHLPP9c groups (*n* = 8 in saline and *n* = 6 in the peptide group). A log-rank (Mantel-Cox) test using Kaplan-Meier curves was performed. *P < 0.01 between saline and TAT-PHLPP9c. (**B**) MAP was measured and analyzed in swine over time (before arrest, during VF, at CPR, and R30 after ROSC). No statistically significant differences were noticed before arrest during VF and CPR for both groups. MAP recovered to 40% of the BL at R30 in the saline group. In contrast, MAP recovered to an average of 92% of the BL level at R30 in the peptide group. Data are represented as mean ± SD. (**C**) Neurologic outcomes improved in the TAT-PHLPP9c group (*n* = 6), with 4 swine being completely intact, 1 not intact, and 1 dead while none of the 8 animals in the saline group survived (*n* = 8). Paired, 2-tailed *t* test was used. *P < 0.01 between saline and TAT-PHLPP9c. Good function was defined as a score ≤ 1 (0 = normal; 1 = mild to moderate lethargy, ambulating and eating; 2 = severe lethargy, ambulating and eating; 3 = awake, ambulating, not eating; 4=awake, not ambulating; 5 = coma; 6 = dead).

We next tested the efficacy of TAT-PHLPP9c in a swine ventricular fibrillation (VF) model. Arrest durations between groups were not statistically different, with the average duration trending less in controls (4.6 versus 4.9 minutes, *P* = 0.68). Five of 6 peptide-treated swine achieved ROSC, whereas 1 of 8 control swine had ROSC (*P* < 0.01). Further, no swine in the saline group lived to 24 hours after ROSC, but 5 out of 6 swine survived to 24 hours (*P* < 0.01) (Figure 7A). As depicted in Figure 7B, there was no statistical difference in MAP between groups at BL (before arrest) or during VF and CPR. MAP recovered to 40% of the BL (36.1 mmHg versus 80.2 mmHg at prearrest BL) in the control swine at R30 as compared with an average MAP recovery of 92% of BL (67.7 mmHg versus 73.7 mmHg before arrest) at R30 in the treatment group. Neurologic outcomes favored the treatment group, with 4 swine being completely intact at 24 hours and 1 lethargic relative to none of the control swine surviving (Figure 7C). Treatment group swine studies were discontinued prior to achieving the control group sample size because survival differences were already dramatic and animal welfare was prioritized.

### TAT-PHLPP9c decreased the release of taurine and glutamate into blood.

**Figure 8:**
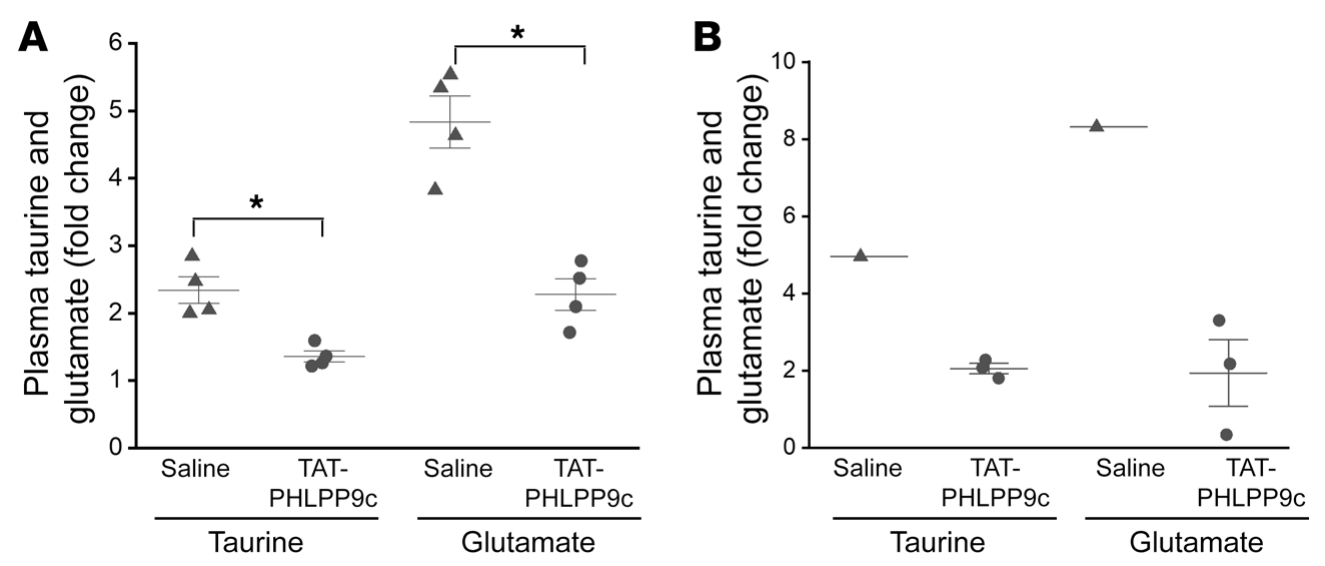
Plasma taurine and glutamate concentrations after CA in mouse and swine. (**A**) TAT-PHLPP9c decreased plasma taurine and glutamate concentrations at R15 after ROSC in mice. Paired, 2-tailed *t* test was used. *P < 0.01 between saline and TAT-PHLPP9c. Data are represented as mean ± SD from 4 mice for each group. (**B**) There was a statistically insignificant difference of decreased plasma taurine and glutamate levels detected as early as R5 after ROSC in the TAT-PHLPP9c–treated swine group (*n* =1 in control, as only 1 swine achieved ROSC; *n* = 3 in the peptide group).

Taurine and glutamate are amino acids expressed primarily in heart and brain, respectively, and are released in response to ischemic injury. Under normal conditions, taurine and glutamate are present in high concentrations in heart and brain tissues and very low concentrations in plasma. They are released from cells with osmotic (as can occur with sorbitol generation), chemical, and mechanical stresses and ischemic injuries (17, 18), such as myocardial infarction and acute stroke. Therefore, plasma taurine and glutamate concentrations were measured in both mice and swine. In the mouse model, compared with the saline group, plasma taurine and glutamate concentrations were decreased by TAT-PHLPP9c measured at R15 (*P* < 0.01) (Figure 8, A and B). In swine, compared with the saline group (only 1 swine achieved ROSC), there was a statistically insignificant difference of decreased plasma taurine and glutamate detected as early as R5 in the TAT-PHLPP9c group.

## Discussion

This study illustrates the design and efficacy of a peptide, TAT-PHLPP9c, for treating CA that mimics mechanisms of CPR cooling without the need for physical cooling. TAT-PHLPP9c can quickly reach tissues of vital organs such as heart and brain within minutes after intravenous injection. It enhanced AKT activation, increased PDH activity, reduced sorbitol generation, and promoted glucose oxidation and bioenergetic recovery after CA. TAT-PHLPP9c also increased cerebral blood flow and cardiac function, as measured by MRI and echocardiogram. Furthermore, TAT-PHLPP9c improved neurologically intact survival after CA in models of mouse asystole and swine VF when administered intravenously during CPR.

The TAT protein transduction domain is a very promising tool for noninvasive cellular import of cargos and has been used in delivery of many therapeutic agents for treating cancer and pulmonary fibrosis (19, 20); it also appears promising in the emergency care setting for acute stroke (21). It mimics cooling protection during CPR that is mediated by AKT activation (9, 10). Strategies to enhance AKT signaling include either increasing the activity of AKT by activators or inhibiting the activity of phosphatases that negatively regulate AKT. This study focused on the latter strategy. TAT-PHLPP9c contains 9 amino acids of the PDZ-binding motif of PHLPP1 phosphatase (1 of 3 major AKT phosphatases, along with PTEN and PP2A), so it is expected to specifically compete with endogenous PHLPP1 at its membrane-binding site without interfering with the ATP-binding activity of PHLPP1. Our results showed that TAT-PHLPP9c completely blocked the interaction of the endogenous PHLPP1 to its membrane adaptor protein NHERF1 at 10 μM while control TAT did not, thereby preventing PHLPP1 from dephosphorylating AKT. In contrast, the PH domain of PHLPP 1 is necessary for the effective dephosphorylation of PKC, another major target of PHLPP1. Our data demonstrated that TAT-PHLPP9c did not affect the phosphorylation of Ser660, one of the phosphorylation sites shared by a number of PKC isoforms, such as PKCα, -β1, -β2, -δ, -ε, -η and -θ (22), which is the main phosphorylation site of PKC regulated by the PHLPP1 PH domain (23). Therefore, this approach in design improves specificity and efficacy of this peptide in order to achieve the desired results and reduces the side effects often caused by small molecule inhibitors that inhibit phosphatase activity by acting on the ATP-binding site common to many phosphatases.

AKT plays a key role in cell survival and metabolic recovery after I/R injury as seen in CA. Our previous work has shown that activation of AKT by cooling inhibits apoptosis and necrosis after ischemic insults in cardiomyocytes (10). Genetic deficiency of *Akt1* abolishes cooling protection and limits downstream targets that regulate metabolism, inflammation, and cardiac contractile function, further supporting a critical role of AKT in CA resuscitation (9). Cooling increases PDH activity in *Akt1^+/+^* mice, but this increase was lost in *Akt1^+/–^* mice, suggesting that AKT mediates PDH activity. Following CA, inactive PDH uncouples glycolysis and glucose mitochondrial oxidation and causes lactate accumulation and acidosis and insufficient bioenergetic recovery (i.e., low ATP content). The proton load (H^+^) resulting from uncoupling of glycolysis and glucose oxidation following CA may account for both the depressant effects on mechanical contraction and Ca^2+^ overload (24) of the heart. TAT-PHLPP9c activated AKT, upregulated PDH activity (decreased PDH phosphorylation), and increased ATP contents in both heart and brain (Figure 4 and Figure 5), confirming that TAT-PHLPP9c improves glucose oxidation and metabolic recovery in vital organs following CA. The control of PDH phosphorylation is regulated by 4 different PDH kinases (PDK1–4) and 2 different PDH phosphatases (PDP1 and PDP2), which are all differentially expressed in tissues (25). PDKs phosphorylate PDH, but PDPs catalyze the reverse reaction. We have measured total PDK4, as it is expressed in both heart and brain, and observed no changes with the treatment of the peptide. Thus, the involvement of other PDK isoforms and PDK-independent pathways, such as AKT/p38/FOXO/PDH and AKT/GSK3β/PDH signaling pathways as reported previously (26, 27), highlights the need for additional studies.

The enhanced coupling of glycolysis and glucose oxidation by TAT-PHLPP9c also reduces glucose diversion to sorbitol generation (Figure 5), a metabolic event related to impaired glucose oxidation, to mitigate the osmotic stress resulting from CA and inhibit taurine and glutamate release from tissues. Because taurine and glutamate are not easily replenished during the early phase after CA, inhibition of their release after resuscitation, within minutes after CPR, could greatly improve the condition of myocardial stunning and brain injury (28–30). The decreased plasma taurine and glutamate correlates with improved survival and neurological function in mice and swine (a trended decrease by the peptide treatment in swine, as only 1 swine in the saline group achieved ROSC), indicating the relevance of plasma taurine and glutamate as possible markers of injury after CA and relevant targets affected by peptide treatment. This finding is supported by a recent clinical study demonstrating that plasma taurine at the time of hospital arrival after out-of-hospital CA is associated with increased mortality (31). In addition to their potential use as biomarkers for predicting CA outcome, taurine and glutamate possess important cytoprotective properties as intracellular antioxidants, mediators of energy metabolism, and regulators of gene expression as well as acting as inhibitory neuromodulators (32–34). Their release into the extracellular space could negatively affect these important intracellular cytoprotective roles.

**Figure 9:**
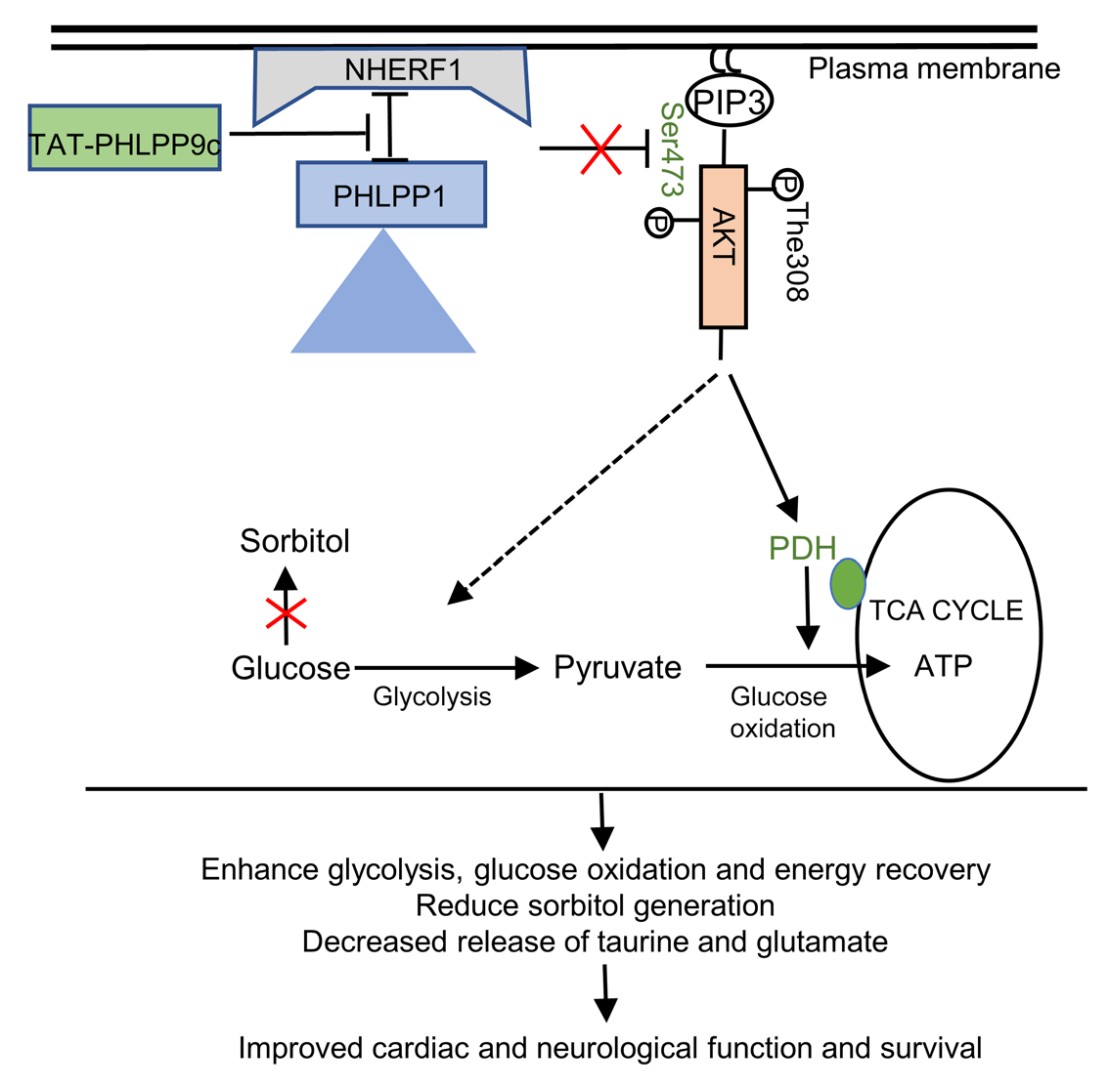
Schematic of hypothesis. TAT-PHLPP9c administration during CPR following CA inhibits the binding of endogenous PHLPP to its membrane adaptor NHERF1. This prevents AKT-Ser473 dephosphorylation, thereby enhancing PDH activity, reducing diversion of glucose to sorbitol via the polyol pathway, leading to increased glucose utilization and bioenergetics recovery and improved heart and brain function and survival.

Longitudinal MRI results revealed that the TAT-PHLPP9c treatment increased rCBF in treated mice compared with control mice at all time points of measurements for up to 2 hours after ROSC. This demonstrated that TAT-PHLPP9c can facilitate the recovery of blood perfusion in brain and serve as a neuroprotective agent after ROSC. rCBF MRI signal is a widely used and noninvasive MRI indicator that is proportional to the physiological CBF (35–37). Although the drug demonstrated consistent and beneficial effects for all the time points up to 2 hours after ROSC, significant rCBF increases with peptide treatment were observed only in early time points, potentially due to limited sample sizes. TAT-PHLPP9c appears to raise rCBF in the whole brain. No specific brain structures affected most were identified because higher spatial resolution with increased signal averaging and longer scanning time would be required, which would reduce temporal resolution for longitudinal studies as a trade-off. The improved rCBF could be a result secondary to improved cardiac function, but our results imply that TAT-PHLPP9c may have a direct protective role in brain. The summary of proposed mechanisms of TAT-PHLPP9c protection is illustrated in Figure 9.

Early delivery of CPR plus defibrillation within 3 to 5 minutes of collapse can increase ROSC rates as much as 49% to 75% (38–40). However, the neurologically intact survival rate is still poor due to impaired metabolic recovery of vital organs, brain dysfunction, and overwhelming systemic inflammation after arrest. TTM using active cooling is thought to be beneficial in post-CA care for neuroprotection. Its optimal timing, duration, and target temperature remain matters of debate, with ongoing concern that cooling protection initiated too late may be less effective. The TAT-PHLPP9c peptide demonstrated a marked improvement of cardiac function, brain perfusion, and function, presenting an opportunity to pharmacologically produce protection without the need to physically cool or to serve as an adjuvant to TTM to achieve a synergistic effect. This study provides evidence that TAT-PHLPP9c, when given during CPR, may stand alone as an effective therapy for CA. Current cooling practices include surface cooling and invasive cooling devices, which require hours to achieve target temperature. Because these techniques involve physical cooling and heat transfer is time dependent, it commonly takes hours to achieve target temperatures of 32°C to 36°C. Cold saline during CPR can be useful, but the resulting decrease in coronary perfusion pressures and pulmonary edema may be harmful. Recent work studying CPR cooling using evaporative nasal cooling failed to demonstrate significant cooling prior to hospital arrival or treatment benefit (41). Hence, the systemic administration of TAT-PHLPP9c via intravenous injection may overcome time limitations and adverse effects of physical cooling. Such a therapeutic strategy also introduces the possibility that the benefits of peptide-induced protection become available to more out-of-hospital and in-hospital patients with different underlying rhythms and for other diseases that involve myocardial and brain I/R injury. Furthermore, in the current clinical practice, several drugs, including epinephrine, lidocaine, and amiodarone, have been used during CPR to improve ROSC, but none of them have a proven benefit on long-term survival and preservation of neurologic function. TAT-PHLPP9c demonstrates promise in animal models for improving ROSC and long-term neurologically intact survival.

Regarding side effects, the relatively small size of TAT-PHLPP9c (20 amino acids) likely limits immunogenicity compared with large protein-based drugs. This feature reduces immunogenicity-induced undesirable effects, such as anaphylaxis and reductions of efficacy. The immune responses, such as flushing, urticarial, and transient hypotension, were not noticed in mouse or pig suggesting that TAT-PHLPP9c has a low immunogenicity.

### Limitations.

This study did not examine whether this peptide is synergistic or additive to cooling therapy in cardiac and neurological protection; this will be addressed in future studies. TAT-GFP, but not TAT-PHLPP9c, was shown to rapidly reach mouse heart and brain tissues, given the limitation of available antibody and detection methods. We are currently developing a protocol that would allow us to detect TAT-PHLPP9c in tissues. The dynamics and off-target effects (other than PKC) of this peptide were ascertained and are currently under investigation. In addition, this study did not address whether the neuroprotection of TAT-PHLPP9c is a primary effect or a secondary effect resulting from improved cardiac function. However, TAT-PHLPP9c activated AKT, enhanced PDH activity, reduced sorbitol production, and increased ATP production in brain at 15 minutes after resuscitation. It improved cerebral blood flow after resuscitation and neurological function in addition to reducing glutamate release from brain. This concurrent timing is consistent with a direct beneficial effect on both brain and heart biology and function.

## Methods

### Design of TAT-PHLPP9c peptide and its mechanism of action.

We have designed a cell-permeable, 20–amino acid peptide, TAT-PHLPP9c, that targets the PDZ-binding motif of PHLPP, representing a specific approach that blocks the localization of PHLPP to its membrane adaptor. Nine C-terminal residues of PHLPP1 (LPNYYNTPL) were rendered cell permeant by linking the peptide to the cell-membrane transduction domain of the TAT protein (YGRKKRRQRRR) to obtain a 20–amino acid peptide, TAT-PHLPP9c (YGRKKRRQRRR-LPNYYNTPL-NH2).

### Isolation of mouse cardiomyocytes.

Primary cultures of mouse ventricular cardiomyocytes were prepared from hearts of 1- to 2-day-old neonatal C57BL/6 mice (The Jackson Laboratory) as described previously (10). Experiments were performed on 6- to 8-day cultures.

### Mouse model of sudden CA.

We have previously reported a mouse CA model (5). To increase severity, the mouse CA model used in this study extended the arrest time from 8 to 12 minutes. Retired breeder C57BL/6 female mice (6 to 9 months old, Charles River Laboratories) were anesthetized with 100 mg/kg ketamine and 10 mg/kg xylazine. The trachea, carotid artery, and internal jugular vein were surgically exposed via a midline ventral neck incision. Following exposure, the trachea was then intubated and ventilated. Two polyethylene (PE-10) lines were inserted into a right carotid artery and a left jugular vein. The venous access was used for potassium chloride and drug injection, and the arterial access was used for continuous hemodynamic monitoring. Three ECG needle probes were inserted into the leg subcutaneously for cardiac monitoring. Rectal temperature was monitored and maintained at 37°C ± 0.5°C throughout the surgical preparation. Following 20 minutes stabilization with MAP greater than 80 mmHg and a partial pressure of end-tidal CO_2_ (P_ETCO2_) greater than 35 mmHg, asystolic CA was then induced by intravenous administration of 0.08 mg/g potassium chloride solution for 12 minutes (8 minutes for MRI and echocardiography studies). Mechanical ventilation was restarted. Mice were resuscitated with chest finger compression over 300 times/min at the end of the 12-minute arrest for up to 5 minutes. All mice received a bolus of epinephrine (0.05 mg/kg) after 90 seconds of CPR along with either TAT-PHLPP9c peptide or the same volume of saline (control) via intravenous injection. ROSC was defined as the return of sinus rhythm with a MAP greater than 40 mm Hg lasting at least 1 minute.

### Short-term survival and long-term survival studies.

Mice were randomized and blinded to receive saline or TAT-PHLPP9c (*n* = 11 each group) for 4-hour (short term) survival studies. Mice were maintained on mechanical ventilation with hemodynamic monitoring for 4 hours after ROSC. A randomized and blinded study was also performed for a 5-day (long term) survival study (120 hours, *n* = 17 each group). Mice remained on mechanical ventilation with hemodynamic monitoring for 2 hours until they showed evidence of spontaneous breathing along with adequate MAP (>55 mmHg). At this time, mice were disconnected from the ventilator for a breathing trial. Once it was determined that the mouse was able to breathe on its own, the mouse was extubated and all vascular access devices were removed, vessels ligated, and surgical wounds sutured. Prior to awakening, mice received a dose of buprenorphine (0.1 mg/kg) for pain control. Mice were placed in a protected recovery area and visually monitored for 2 hours before they were returned and individually housed in the animal facility for observation twice a day up to 5 days. The neurological function of surviving mice was evaluated with an established scoring system, including level of consciousness, corneal reflex, respiration, righting reflex, coordination, and movement/activity, as reported previously (11, 42). The scores range from 0 (death) to 12 (normal neurological function). When any one of the criteria listed was zero or at the end of the protocol, these mice were euthanized. Mice that experienced technical issues or did not achieve asystole arrest after randomization were excluded from the studies.

### Echocardiography studies.

To study the effect of TAT-PHLPP9c on cardiac function after ROSC, an 8-minute asystole arrest mouse model was used. Mice were randomized into saline and TAT-PHLPP9c treatment groups (*n* = 4 mice each group). Once mice achieved ROSC, they remained on mechanical ventilation and under hemodynamic monitoring until they showed evidence of spontaneous breathing along with an adequate MAP of greater than 55 mmHg. Mice were monitored for 10 minutes before being disconnected from the ventilator for a breathing trial. Once a mouse was determined to be able to breathe on its own, it was extubated and transported to the cardiovascular core for cardiac-function measurement by echocardiogram. Eight mice were randomized into 2 groups (saline and TAT-PHLPP9c, *n* = 4/group) and were imaged using a Vevo 2100 (VisualSonics Inc.) equipped with a MS550 transducer (22–55 MHz). Mice were placed on the warming plate and their body temperatures were maintained at 37°C. They were connected via a nose cone to a vaporizer with 100% O_2_ and 0% isoflurane (mice were anesthetized with ketamine/xylazine). The electrode gel was evenly applied to the 4 paws taped to the ECG electrodes. Two-dimensional mouse heart parasternal SAX, long-axis, M-mode, color-flow Doppler, and tissue Doppler images were captured at R40 and R80 after ROSC for each mouse. Data analysis for CO, EF, FS, and SV, E and A waves, IVRT, and IVCT was performed using Vevo Analytic Software, version 5.7.0 (VisualSonics).

### MRI studies.

The same mouse model and post-ROSC procedures were used to study the effect of TAT-PHLPP9c on brain perfusion after ROSC. Once a mouse was determined to be able to breathe on its own, it was extubated and transported to the nuclear magnetic resonance (NMR) facility for brain MRI measurements of rCBF. Ten mice were randomized into 2 groups (saline and TAT-PHLPP9c, *n* = 5/group) and were imaged with an Agilent Varian 9.4T preclinical MRI scanner with a 39 mm proton volume coil. Body temperature was maintained at 37°C by regulating the warm airflow into the scanner bore. Temperature and respiration rate were monitored using an MRI-compatible physiological monitoring system (model 1025, SA Instruments Inc.). Longitudinal arterial spin labeling (ASL) MRI was performed every 20 minutes for each mouse for up to 2 hours after ROSC. Due to transportation and imaging setups, the first MRI data set was collected at approximately R40 after ROSC; images were captured at R40, R60, R80, R100, and R120 after ROSC. The ASL MRI was performed on a 1 mm central brain slice using a flow-sensitive alternating recovery (FAIR) ASL sequence with inversion pulses for global or 4 mm slice regions followed by a steady-state free precession (SSFP) centric k-space readout (43). Other imaging parameters included the following: inversion times (TIs) = 0.1, 0.25, 0.5, 0.75, 1, 1.25, 1.5, 1.75, 2, 2.5, 3, 3.5, 4, 6, 8 seconds; readout echo time (TE)/repetition time (TR) = 0.9/1.9 ms; overall TR = 2.5 seconds; field of view = 20 × 20 mm^2^; and matrix size = 128 × 128 pixels. An image without inversion was also collected as reference. FAIR ASL MRI data sets were processed using a phase-adjusted bipolar T_1_ recovery fitting to both global and slice-selective inversions (43). Percentage MRI contrast proportional to the physiological CBF, the rCBF, was calculated based on 100 × (*S_ss_* – *S_gs_*)/*S_noi_*, where *S_ss_*, *S_gs_*, *S_noi_* are the signals from the slice, global, and no-inversion acquisition, respectively. rCBF contrast maps were color coded and overlaid over the corresponding anatomical T_2_-weighted MRI images. Whole-brain percentage rCBF signals were averaged and compared at each time point between saline and TAT-PHLPP9c groups.

### Western blot and immunoprecipitation analysis.

TAT-GFP was used to measure the kinetics of TAT-linked protein delivery to heart and brain tissues. Mice received intravenous injection of 7.5 mg/kg of TAT-GFP. The heart and brain were collected at various times (0, 5, 15, 30, and 60 minutes) for detection of TAT expression by Western blot analysis using GFP antibody (SAB5701259, MilliporeSigma). For other studies, mouse heart and brain tissues were harvested at R15 after ROSC, snap-frozen in liquid nitrogen, and stored at –80°C. Samples were pulverized, lysed in lysis buffer, and then analyzed using a standard protocol for Western blot (9). Protein phosphorylation and expression were detected with antibodies against p-AKT-Ser473 (catalog 9271, Cell Signaling Technology), GSK3β-Ser9 (catalog 9336), and PKCβ2-S660 (catalog 9371), α-tubulin (catalog 2144), AKT (catalog 9272), and GAPDH (catalog 2118), phosphorylated PDH E1-α subunit p-Ser293 (catalog NB-110-93479, Novus Biologicals), and β-actin (catalog A5441, MilliporeSigma). Quantitative results were obtained via densitometry (ImageJ, version 1.42, NIH). For immunoprecipitation experiments, 500 μg of mouse brain lysates (1 mg/mL) were preincubated with 1 and 10 μM of TAT-PHLPP9c or 10 μM TAT for 30 minutes, followed by immunoprecipitation with 5 μL of anti-NHERF1 antibody (catalog ab135957, Abcam) and 30 μL of protein A/G-sepharose bead for 2 hours, and the precipitated proteins were detected by PHLPP1 antibody using Western blot analysis.

### Immunohistochemistry of brain and heart.

TAT-GFP was also measured by immunohistochemistry analysis to detect the localization of the peptide 1 hour after intraperitoneal injection. Brain and heart tissues were fixed and sections were properly handled and stained with 1:1,000 rabbit polyclonal anti-GFP antibody as reported previously (13). The reaction color was developed by treating tissue sections with DAB substrate (DAB Substrate Kit, Vector Laboratories). The sections were washed with water, counterstained with hematoxylin, dehydrated, and mounted with VectaMount Permanent Mounting Media (Vector Laboratories). Slides were then examined under a microscope with ×200 magnification using Smart Capture VP imaging software, version 13.1.

### ATP measurement.

ATP concentrations in mouse heart and brain at R30 were measured with a colorimetric ATP Assay Kit (Abcam) according to the manufacturer’s instructions.

### Swine model of VF CA.

Fourteen adult female Yorkshire pigs (30–35 kg, Archer Farms) were injected with 22 mg/kg ketamine and mechanically ventilated in the supine position with 100% O_2_ and 1% to 2.5% isoflurane. Via 8 French sheaths, access to both femoral veins and arteries was acquired. Pig-tail catheters were placed in the descending thoracic aorta and right atrium for continuous measurement of arterial and central venous pressures, respectively. External defibrillator electrodes were placed on both sides of the chest. After cessation of isoflurane and mechanical ventilation, VF was induced by electric shock via defibrillator electrodes. Fibrillation was allowed to persist for an average of 5 minutes before initiation of advanced cardiovascular life support (ACLS). Mechanical ventilation and simultaneous vest CPR (44) at an inflation pressure of 200 mmHg, 60 cycles per minute, and a 40% duty cycle were performed. Two doses of 7.5 mg/kg each of TAT-PHLPP9c (it usually required 2 doses of the peptide for swine to achieve ROSC) or the same volume of saline was administered during CPR at 1 and 3 minutes of support along with 0.5 mg/kg epinephrine boluses to either control or the TAT-PHLPP9c group via the venous line. After 3 minutes, shocks were discharged to restore sinus rhythm. ACLS was continued until ROSC. Hemodynamic data were recorded at pre-arrest, during VF and CPR, and after ROSC, including MAP. Plasma samples (2 mL) were taken at BL, R2, and R30 after ROSC. Survival and neurologic outcomes were then quantified on the modified Rankin scale (45), adapted for swine. Swine were recovered for 24 hours after anesthesia, and a neurologic score was assessed based on overall physical well-being. Good function was defined as a score less than or equal to 1 (0 = normal; 1 = mild to moderate lethargy, ambulating and eating; 2 = severe lethargy, ambulating and eating; 3 = awake, ambulating, not eating; 4 = awake, not ambulating; 5 = coma; 6 = dead). Swine were euthanized via KCl injection under isoflurane and proper sedation at the end of the protocol.

### Measurement of plasma taurine and glutamate concentrations.

Mouse blood samples were collected from the same mice used for molecular analysis at R15 and collected at R5 from swine. Samples were collected into EDTA tubes and centrifuged within 60 minutes at 2,000*g* for 10 minutes, and plasma was obtained and used for taurine and glutamate measurements using commercially available assay kits (Abcam).

### Statistics.

Results were presented as mean ± SD for continuous data or frequency and percentage for nominal/ordinal data. One-way ANOVA was followed by Dunnett’s test for multiple comparisons between control and others. Student’s *t* test (2-tailed) was used to compare means of 2 groups. A χ^2^ test was utilized for the ROSC rate comparison. For survival analysis, a log-rank (Mantel-Cox) test using Kaplan-Meier curves was performed. Based on the power with effective size and our previous experiences, *n* = 11 mice for each group and *n* = 17 mice for each group were used for a 4-hour survival study and a 5-day survival study, respectively. A *P* value of less than 0.05 was considered significant. All analyses were performed using SAS, version 9.4.

### Study approval.

All animal procedures were approved by the Institutional Animal Care and Use Committees of the University of Illinois at Chicago and Johns Hopkins University and performed according to the *Guide for the Care and Use of Laboratory Animals* (National Academies Press, 2011).

## Author contributions

JL designed research studies, conducted experiments, acquired data, analyzed data, and contributed to writing the manuscript. XZ designed research studies, conducted experiments, acquired data, analyzed data, and contributed to writing the manuscript. MTO conducted experiments, acquired and analyzed data, and drafted methods and results for swine studies. CL, SL, CNJ, KQ, and AWB conducted experiments and acquired data for mouse studies. SJF conducted experiments and acquired data for swine studies. FCD conducted experiments, acquired data, and analyzed data for MRI studies. HK analyzed data. JC collected data and analyzed data for echocardiography. KC designed MRI studies and drafted methods and results for MRI studies. HRH designed swine studies. TLVH designed research studies, contributed to writing the manuscript, and oversaw the entire project. JL and XZ contributed equally to study design and data acquisition. JL contributed to data analysis, writing, and revising the manuscript.

## Supplementary Material

Supplemental table 1

## Version 1 05/01/2023

Electronic publication
