# Supplementary material for: A cell-penetrating PHLPP peptide improves cardiac arrest survival in murine and swine models
Source: J Clin Invest. 2023 May 1;133(9):e164283. doi: 10.1172/JCI164283 (PMC10145924; doi:10.1172/JCI164283)
Supplement: Supplemental table 1 [file jci-133-164283-s057.pdf]

Supplemental Table 1. Comparison of cardiac function between saline and TAT-PHLPP9c

Cardiac Function	SAX R40		*p*-value	SAX R80		*p*-value
	Saline (n=4)	TAT-PHLPP9c (n=4)		Saline (n=4)	TAT-PHLPP9c (n=4)	
Cardiac Output (CO, mL/min)	3.1762<br>4.9782<br>7.1690<br>4.4492	8.0087<br>6.8827<br>6.3788<br>5.1163	0.158	4.6025<br>4.8830<br>6.0149<br>5.6820	9.8433<br>10.9209<br>5.9417<br>7.0520	0.041
Ejection Fraction (EF, %)	17.2854<br>31.7308<br>34.3617<br>23.8008	45.5230<br>29.2304<br>40.7749<br>32.4477	0.108	22.6429<br>30.9510<br>28.6417<br>32.9387	49.9397<br>40.6821<br>38.2792<br>38.9100	0.01
Fractional Shortening (FS, %)	7.5482<br>14.5426<br>16.0029<br>10.7864	22.0584<br>13.4730<br>19.5246<br>14.8945	0.105	10.0938<br>14.0260<br>14.7025<br>15.2320	25.2861<br>19.6259<br>18.0874<br>18.4670	0.015
Stroke Volume (SV, µl)	8.5978<br>15.3045<br>20.3818<br>13.8142	23.3741<br>20.7533<br>19.0125<br>14.0006	0.179	13.3745<br>11.9360<br>17.8507<br>17.2828	30.5775<br>28.6710<br>21.0368<br>22.5620	0.008

Cardiac systolic function including cardiac output (CO), ejection fraction (EF), fractional shortening (FS) and stroke volume (SV) were measured by echocardiogram using parasternal short-axis (SAX) M-mode images of cross-sectional view of left ventricles captured at 40- and 80- min post-ROSC (SAX R40 and SAX R80) in saline and TAT-PHLPP9c-treated mice. n=4 each group. $p < 0.05$ between saline and TAT-PHLPP9c at R80.
